# Serum Therapy1Read for St. Luke’s Clinical Society.

**Published:** 1912-05

**Authors:** W. S. Garlock

**Affiliations:** Little Falls, N. Y.


### Serum Therapy[1]

By DR. W. S. GARLOCK
Little Falls, N. Y.

THE subject treated in this paper is not a new one and the past year's work comprises but an extension of former work but it may not be inopportune to try to take a general glimpse of serum therapy and to try to see where it stands in the treatment of disease and the larger field of biology. The experimental work on which it rests has given most marvelous

1. Read for St. Luke's Clinical Society.

revelations of the complexity and interdependence of the various functions and cells of the body.

A correct idea of this subject depends largely upon a knowledge of the physiology of the component cells of the body.

We may premise by saying that an animal is a republic of cells each with its own life and metabolism. Let us for a moment consider one of the simplest animal cells that lives an independent life.

The amoreba is nothing, but a single celled animal and in that one cell we find all the functions of cellular life—elementary and undifferentiated. It grows, eats, digests, assimilates, exerts, moves, reproduces its kind and has enough feeling or sensation to know what particles it comes in contact with are suitable for food. In a large way this is a type of animal cell to which even the differentiated cells of our own bodies are no exceptions as we can simply by a little reflection perceive that these are all functions necessary to cell life. The ovum is a single cell that after fertilization undergoes segmentation at first simply but in a short time the various cells begin to take on peculiarities due to differentiation but this is largely a development of one of the primordial functions of the simple cell. For instance, the motor functions of the simple cell become more and more prominent until it finally develops into a muscular cell but still retains in diminishing degree all of its primitive functions that are necessary to its cell life. So with all the other peculiar cells, some becoming glandular cells, some nerve cells and so on. There must be a harmonious development of all these cells or the various organs finally formed will not be right. All these cells must be nourished by the blood and all of them must get rid of their excretions. Some of the excretions of the cells are simply to be removed from the body republic of cells while other excretions are of use to other cells either as food or as stimulants to their function. These latter are the internal secretions and hormones as they have been called.

In the differentiation of the cells in the body development the various kinds of cells have become differently susceptible to influences of environment and attack from organized and unorganized substances in the physiological atmosphere in which they live. They also need different food elements on which to live and from which they get their functional energy. Thus one class of cells will take from the lymph and blood what they need and after using it in their metabolism pour their excretions which in some cases are secretions back into the lymph and blood. These excretions are removed in one way or another either directly or through the metabolism of other cells with excretory functions. If these excretions are attained unduly in the system they are disease producing. The secretion of these cells alluded to that are prepared for the use of other cells either act as food for other cells or act as stimulants or regulators of their action. Thus an excess of the secretions of the thyroid gland act on the heart and nerves to case Graves' disease while their deficiency causes cretinism. The adrenals islands of Langerhans, thymus hypophysis, etc., produce such active secretions. The relationship of the different species of the animal kingdom is so close that such cell products of one animal in some cases can be used to modify the cellular activities in man and other animals and on these foundations is being built up organo-therapy.

In the body republic or the individual man certain cells are differentiated into reproductive cells as ova and spermatozoa. These cells carry the hereditary principles capable of development into new animal republics with all the developmental potentialities needed for this kind of reproduction. Most of the separate differentiated cells of the body retain their reproduction capacity so far as it is needed to reproduce their own kind of cells but in some cases this seems to be lost at least ordinarily and certain tissues are largely made up of mother cells producing daughter cells that have a function but do not reproduce their kind but their functions are terminal. Thus the blood cells do not ordinarily reproduce but are daughter cells of the mother cells in the blood-cell-making organs and tissues. These cells do their work and die. Their production seems to be almost unlimited. In a large measure they are the defensive and reparative cells that defend the animal against infections of various kinds. Thus the white blood cells are in a way the scavengers of the system and eat up the foreign organisms that get into the body republic. They are the policemen and executioners of the animal republic. This function while probably largely exhibited by the white blood cells is not confined to them but remains more or less in the various tissue cells.

These defensive cells are able in some cases in one way or another to protect the body against toxic or disease producing substances or organisms accidentally getting into the body republic and they do this in various ways either by phagocytosis or the formation of various antibodies which remain in the cells and body fluids for a longer or shorter time and while there more or less perfectly protect the cell republic against the foreign element in the body politic.

The productive action and use of the antibodies so formed and the various modifications of the body cells and fluids resulting from the activities of these foreign elements in the body open the broad field for the study and science of immunity and the cure of infectious diseases.

The body is protected against disease by various lines of defenses. First, by sanitation and health laws. Second, by the skin and mucous membranes, including the digestive juices; third, by the blood and body fluids and by the cells first of the blood and later by the tissue cells. The blood being the general medium by which foreign substances are distributed in the body, its white cells constitute the skirmish line of the defensive forces.

A study of the invading forces becomes interesting at this point and we may premise by saying that these forces may be organic or inorganic, living or dead and a complete consideration of them would include drugs as well as all toxins but we shall in following remarks confine ourselves to toxins as that is the particular part of the therapeutic frontier we wish to consider.

Let us first consider *toxins*. These are products of all life and are either free toxins which correspond to excretions of their producing cells and are cast off to be removed by the physiologic atmosphere in which they live and exert their malign influence immediately or are *endotoxins* which are probably like the internal secretions of our own organs and do not enter the fluids of the host until the cells producing them are dead and disintegrated.

The toxins may get into the body either by the cells that produce them getting into the body and producing the toxins after their entrance or they may be produced outside of the body and get into the body of the host in various ways. Some of the cells introduced quickly succumb to the body defenses in one way or another like the influenza germs or else they may live on indefinitely like the germs of tuberculosis, syphilis, etc.

Some germs seem to leave a lasting impression on the body so that one infection protects against subsequent ones like smallpox while others seem to rather predispose the host to subsequent attacks like influenza. It goes without saying that the invading hosts vary in their toxicity and destructibility and it is entirely probable that the products of some foreign cells are beneficial because they supply the body with useful substances or they have been a part of human environment for so many generations that we have become immune to them and are adapted to their influence. In this way the various species of animals are immune to or but slightly affected by these germs and toxins some to one and some to another and so on and the discovery of what makes one species immune to a toxin and the possibility of using that protective something or antibody obtained from an immune animal to protect or cure one that is not immune is a great object in experimental work and the work going on in these lines is very promising not only in the protection against infection but in the cure of disease. Passive immunization is arrived at in this work.

Protection against and cure of diseases caused by the entrance of germs may be due to the leucocytes eating the intruders as in phagocytosis and in some cases the efficiency of this process may be increased by opsonin treatment which rather seems to affect the germs to make them subject to phagocytosis than the leucocytes to increase their appetites and ability to destroy the germs. As the germs become more virulent in nature, they become less subject to phagocytosis. Opsonins seem specific in their action on germs and only act on the species from which they are produced. Another and perhaps more important field of work is that of active immunization where the animal is treated to cause the production of the antibodies in the cells and fluids of the body treated. This is illustrated in vaccination where living germs are introduced or inoculated or when dead germs are introduced to get the effects of the endotoxins or where simply toxins are introduced to get their effect on the body cells.

This is the aim of vaccine and serum therapy.

In some cases the nature of the germs is modified before they are used.

We may now ask the question: How are the various antibodies formed and how do they act? But anything like a complete statement of the knowledge we can get from a review of the literature on this subject would be tiresome and take too much time.

The toxins seem to be organized molecules somewhat of the nature of ferments and a peculiarity of these bodies is that they seem to be composed of two groups or parts of different natures one is the toxiphone group which is the toxic or business partner and the other the haptophore group which has the ability to unite with the cell attached. A familiar illustration of this dual function is the ordinary postage stamp and this seems to be applicable to all the toxins endotoxins and antigens that are so much spoken of in literature.

A postage stamp without any mucilage would not be able to unite and a letter would not be affected by it. A letter without any part that would adhere to mucilage would be immune for instance, an oiled paper. Now, suppose the stamp and letter alive and the stamp becomes adherent. The letter to have the stamp adhere must have a receptor or part where the stamp can adhere or unite chemically. Now suppose the stamp causes the letter to throw off as by a kind of disquamation pieces of paper that are fitted to adhere to the stamp and these pieces of paper float freely. New stamps would adhere to them and not to the letter and if a great many free pieces of paper or receptors are thrown off, so many of the stamps would adhere to them that the letters would not be hurt.

These free receptors are the antibodies that protect the letter and simple antitoxins are of this nature. By inoculaion of horses with increasing doses of diphtheria toxins the horses' blood becomes so loaded with free receptors or antitoxin that when injected into a child with diphtheria toxin in its blood, the toxin unites with the antitoxin and is neutralized.

Now, suppose the pieces of paper thrown off from our attacked letter are not simple pieces of paper but are like postage stamps with an active and a binding group. The binding group unites with the binding side of the toxin and the active part affects it in one way or another. The agglutinins and precipitins are of this order.

Amboceptors are like pieces of paper with two binding groups and serve to unite cells, toxins and complemnets. The complement unites with the complementophile group of the amboceptor and the other group of the amboceptor unites with the cells and in this way through the intermediary amboceptor the complement causes lysis as hemolysis, bacteriolysis, etc. The complements are normally present in the blood or are the products of infection.

If there is an excess of amboceptors in the blood, some of them may unite with the complements in the blood and other amboceptors may unite with the cells so that the complement is fixed because amboceptor cannot unite with amboceptor and so the complements cannot get at the cells. In these cases lysis is not produced but may again be made possible by introducing fresh complement.

The disappearance of free complement in a mixture of antigen and antibody is called fixation of the complement. The condition of the complement in such cases is determined by adding erythrocytes and a specific lytic amboceptor for them; now if the complement has been fixed there will be no hemolysis. This indirect way of testing for antibody or antigen is the basis of Wasserman's test.

Now, if the toxin has its toxiphere group destroyed or modified the haptophore group may still in some cases unite with the cell receptors and stimulate receptor or antitoxin formation. Here the face of the postage stamp is canceled. By passing the toxin-producing germs through animals or by various ways of cultivating them or by physical or chemical treatment the toxic qualities or potentialities of germs may be modified so that they can be introduced into the animal experimented on and antitoxin produced because the haptophore group remains potent when without such modification the inoculation would be fatal. After partial protection germs of greater virulence may be injected and so on in increasing doses until a large amount of antibody is produced. This antibody may, in some cases, be used to protect fresh animals against the toxic agent. After in this way getting passive immunization active immunization may be induced by the use of germs and toxins. This is done not only in the treatment of disease in man and as a protection against disease, but is of great importance in protecting domestic animals against infection.

A few words on anaphylaxis may not be out of place in our sketch. If a guinea pig is injected with a small amount of horse serum, it will not seem to suffer but if after a certain period it receives a second injection, it will quickly show signs of inspiratory distress and often die quickly. It is then in a state of anaphylaxis toward horse serum. Anaphylaxis seems to be a reaction to proteins rather than to toxins. It seems to be a part of the immunization program of the cells and in this way it has been determined by experiment that when the second injection of horse serum in the guinea pig, for instance, is made the polymorpho nuclears largely leave the systemic circulation and accumulate in the lungs. As F. W. Andrews in the Croomian lectures says they seem to need oxygen to combat the serum invasion and the anaphylactic symptoms are present while the lung is so loaded with the polymorphonuclears. As soon as the cells go back into the general circulation, the anaphylactic symptoms subside. This rush of polymorphonuclears to the lung is marked in immunized animals after the toxic attack. In ordinary infections the entrance of the toxin is slow or at least not in a sudden large amount as in anaphylactic experiments and so immunity is possible. Andrews even proposes the test for leucopoenia after infections as an index of immunity. This would make the anaphylactic curve correspond with the antibody curve. Figuratively speaking, the defenses of the body are sufficient for the ordinary toxic invasions but when a massive invasion occurs, there is a panic and the rush of leucocytes to the lungs for ammunition causes leucopoenia and anaphylaxis. An unfortunate but fortunately rare example of anaphylaxis occurs sometimes in the use of a second dose of diphtheria antitoxin after an interval of two to several weeks. It is of interest to note that these cases are more apt to occur in asthmatics and particularly in those whose asthmatic attacks are brought on by the odor of horses.

The idiosyncracies of people toward strawberries, crabs, egg albumen, etc., may be of an anaphylactic nature. The whole subject is very interesting but hard to understand. It seems to be a panic of the defensive forces but shows that they are present in the body politic of the cell republic.

The writer feels that too much time has already been taken to go into any of the details of serum therapy and that it is better to be content with this superficial sketch. He feels that the whole subject is of most importance and promises great results in the treatment of infectious diseases and in those conditions like arteriosclerosis, gout and other diseased states where some materies morbi may be at the root of things.

The therapeutics of the past has been almost entirely that of the body but now we are looking forward to therapeutics of the cells.

